# Supplementary Light Differently Influences Physico-Chemical Parameters and Antioxidant Compounds of Tomato Fruits Hybrids

**DOI:** 10.3390/antiox10050687

**Published:** 2021-04-27

**Authors:** Onofrio Davide Palmitessa, Miriana Durante, Sofia Caretto, Francesco Milano, Massimiliano D’Imperio, Francesco Serio, Pietro Santamaria

**Affiliations:** 1 Department of Agricultural and Environmental Science, University of Bari Aldo Moro, Via Amendola 165/a, 70126 Bari, Italy; onofrio.palmitessa@uniba.it (O.D.P.); pietro.santamaria@uniba.it (P.S.); 2 Institute of Sciences of Food Production, National Research Council of Italy, 73100 Lecce, Italy; miriana.durante@ispa.cnr.it (M.D.); sofia.caretto@ispa.cnr.it (S.C.); francesco.milano@ispa.cnr.it (F.M.); 3 Institute of Sciences of Food Production, National Research Council of Italy, 70126 Bari, Italy; massimiliano.dimperio@ispa.cnr.it

**Keywords:** dry weight, total soluble solids, titratable acidity, lycopene, vitamin C, carotenoids, light-emitting diodes, horticulture

## Abstract

One of the challenges for agriculture in the coming years will be producing more food avoiding reducing the nutritional values of fruits and vegetables, sources of nutraceutical compounds. It has been demonstrated that light-emitting diodes (LEDs) used as a supplementary light (SL) technology improve tomato yield in Mediterranean greenhouses, but few data have been reported about SL effects on fruit physio-chemical parameters. In this study, three tomato hybrid (F1) cultivars were grown for year-round production in a commercial semi-closed glasshouse in Southern Italy: red cherry type (“Sorentyno”), red plum type (“Solarino”), and yellow plum type (“Maggino”). From 120 to 243 days after transplant (DAT), Red/White/Blue LEDs were used as SL. The fruits harvested 180 DAT were analyzed and those obtained under LEDs had 3% more dry weight, 15% more total soluble solids, and 16% higher titratable acidity than fruits grown only under natural light. Generally, the antioxidant activity and the mineral profile of the fruits were not negatively influenced by SL. Lycopene content was unchanged and vitamin C content of “Sorentyno” even increased by 15% under LEDs. Overall, LEDs used as SL technology could be one of the tools used by agriculture in Mediterranean basin to produce more food maintaining high quality production.

## 1. Introduction

After decades of great advances in the global fight against hunger, food insecurity and malnutrition, world population is now witnessing a reversal of the progress made: in the last years, the number of undernourished people has increased to 821 million, taking us back to the figures registered a decade ago [1]. Sustainable agriculture and food systems play a central role in ensuring the (now utopic) aims of ending hunger and malnutrition; in this context, greenhouse horticulture sector is continually improving its technological level to increase yield and nutritional content of products. The technological developments of greenhouse sector do not necessarily solve hunger and malnutrition, especially for the areas where hunger and malnutrition are an everyday problem, but it aims to reduce the environmental impact of horticulture and to increase the healthy properties of vegetables. Moreover, based on the United Nations sustainable development goals, high-tech greenhouses will remain the most efficient systems for food production [1] and the use of artificial lighting allows to increase the vegetable production using the same cultivated area.

Tomato (*Solanum lycopersicum* L.) is one of the vegetable species most cultivated in greenhouses; for this reason its importance as a food has been recognized worldwide [2]. In Europe, Italy, together with Spain, is the leader of fresh tomato production with a cultivated area of 17,000 ha open field and 7610 ha greenhouses [3]. The average production of fresh tomato in Italian greenhouses is around 7–9 kg m^−2^ [3]. At the same time, tomato is the most cultivated species in Netherlands greenhouses with almost 15,000 ha with an average yield of 60–70 kg m^−2^ [2]. The high yields of fresh tomato obtained in Dutch greenhouses is due to the high-technological systems of greenhouses and the crop cycle used. The Dutch growers use to transplant tomato plants in December, harvesting tomato fruits from February to November; the Italian and Spanish growers utilize two crop cycles because of the low technology of the greenhouses that makes it difficult to produce high yields while facing extremely hot climate conditions (July–August) and low global radiation levels (December–January). So, the harvesting period of tomato fruits is shorter in Mediterranean basin than in Netherland, contributing to obtain low yield in this area.

One of the technology systems installed in Dutch greenhouses to improve tomato yield is supplemental light (SL) [4]. With SL it is possible to increase the natural daily light integral (DLI), especially during fall-winter season, keeping constant the tomato production all year-round [5]. Currently, high pressure sodium (HPS) is the most used SL technology in the commercial greenhouses, but the introduction of light emitting diodes (LEDs) has received a great deal of attention in the past decade [6]. Recently, there is a great interest on LEDs used as a source of artificial light for greenhouse horticulture in Mediterranean basins [7]. In Southern Italy, because of low DLI integral levels during winter period, it is common to arrest tomato cultivation during this time interval, thus reducing the annual yield [8]. On this regard, Palmitessa et al. [9] reported that using LEDs as SL source in a Mediterranean semi-closed greenhouse it is possible to increase by more than 25% the tomato yield. At the same time, it is important to maintain fruit quality and nutritional values. In fact, tomato is considered a “functional food” [10], because it is rich in antioxidant compounds and mineral elements and it is an important source of bioactive compounds [11].

Carotenoids are compounds to which health-protective properties are attributed. Tomato is the greatest carotenoid source for the population worldwide [12]. Lycopene is the most abundant carotenoid found in red tomato varieties, predominantly in the all-[E] isomer conformation, with high antioxidant activity [13,14]. Furthermore, lycopene is thought effective in preventing some types of cancers and modulating immune and inflammatory responses [15]. Finally, vitamin C or ascorbic acid is the most abundant water-soluble antioxidant found in plants; for this reason, those who follow a vegetarian diet easily reach the recommended daily amounts of 100 mg; however, some circumstances of infection or pregnancy require higher quantities [16,17]. Vitamin C is a cofactor for many enzymes and it has a central role in cell division and growth, in programmed cell death, photosynthesis, iron uptake, as well as in defense response against biotic and abiotic stresses [18]. Vitamin C cannot be produced by humans and it is primarily assumed by vegetables and fruits. It has been shown that genotype [19], moderate salt stress [20], and potassium level in the nutrient solution [21,22] influence the content of antioxidant molecules, but even varying some environmental factors, as light spectra and intensity, it is possible to improve antioxidant content of tomato fruits [23].

Several studies were conducted in northern greenhouses on the influences of LEDs on the chemical composition of tomato fruits. LEDs enhances the antioxidant level of tomato fruits [24]. On this regard, it has been shown that red (R) + blue (B) supplemental light promote lycopene synthesis in tomato fruits [25], while white (W), R, and B LEDs increased the total soluble solids (TSS) content, but did not increase the vitamin C content [26]. Instead, R+B LEDs did not change potassium (K^+^), calcium (Ca^2+^), magnesium (Mg^2+^), TSS, and titratable acidity content but increased sodium (Na^+^) content of tomato fruits [27]. Finally, Ouzounis et al. [28] found that R and B LEDs increase the phenol content of tomato fruits and the effects of SL on the quality of tomato fruits was genotype-specific.

In our previous research [9] it was found that R + W + B LEDs increased the yield of three tomato hybrids grown in an innovative commercial semi-closed greenhouse in Mediterranean basin. This work is a direct continuation of the previous one and aims at obtaining more insights into the effects of the SL system of the fruits of three tomato commercial typologies (cherry, red plum, and yellow plum) selected among those already grown in the crop cycle conducted in Palmitessa et al. [9]. In particular, the ability of LEDs toplight to preserve and/or improve the nutritional values and the chemical composition of the selected tomato hybrids has been here investigated.

## 2. Materials and Methods

### 2.1. Experimental Set-Up

The experiment was performed from August 2019 to April 2020 in a commercial semi-closed glasshouse placed in Monopoli (BA), Italy (40.9027253 N, 17.3277492 E). The height of the gutter of the glasshouse is 7 m, while the maximum height is 8 m. The glasses installed for the roof of the glasshouse are Albarino Low Haze 2AR (Saint-Gobain, Courbevoie, France), with 96.5% of light transmission measured with the Normal (NEN 2675) method.

Light treatments (LEDs + natural light and only natural light) were separated into two different glasshouse compartments (with three blocks (replicates) to avoid all possible negative interaction between them (i.e., shadowing, microclimate, pests, and disease outbreaks). More detailed information about experimental set-up has been described by Palmitessa et al. [9].

### 2.2. Plant Materials and Growing Conditions

Three hybrids (F1) of tomato (*Solanum lycopersicum* L.) were tested: a red cherry—“Sorentyno” (RC; Gautier)—and two plum tomatoes, one red—“Solarino” (RP; Rijk Zwaan)—and one yellow—“Maggino” (YP; Rijk Zwaan).

The seedlings were obtained from a commercial nursery and transplanted on rockwool slabs (Grodan Vital, 100 × 20 × 7.5 cm) on the 23rd of August 2019. Plant density was 4.73 stems m^−2^ and plants were trained vertically and topped 238 days after transplant (DAT). The rockwool slabs were placed on metal gutters (length 100 m, width 0.20 m, 0.15% sloped). Crop operations were described by Palmitessa et al. [9]. During the experimental activity, greenhouse day temperature was 22.5 ± 2.32 °C, while night temperature was 17.7 ± 2.21 °C; the average 24 h relative humidity was 67% ± 0.05% and the average CO_2_ concentration during the day was 482 ± 77.52 mg L^−1^. Bumblebees (*Bombus terrestris* L.) guaranteed the pollination, while pests control was made using integrate pest management systems [9].

The water quality used for nutrient solution (NS) preparation was ranked as 1 [29]; in fact, the electrical conductibility (EC) was <0.7 mS cm^−1^, while Cl and Na concentrations were 16.1 and 31.5 mg L^−1^, respectively. Three different NS were used according to the plant stage [9], but during the light treatment the NS composition was kept constant at these levels (expressed in mg·L^−1^): 124 N-NO_3_, 5 N-NH_4_, 300 K, 41 P, 12 Mg, 94 Ca, 19 Cl, and 47 S. Micronutrient concentration was the same throughout the growing cycle, according to Hoagland and Arnon [30]. More detailed information about NS management and fertigation schedule are described by Palmitessa et al. [9].

The harvest started between October and November (depending on the cultivar).

### 2.3. Supplemental Light Treatment and Daily Light Integral (DLI)

The supplemental light (SL) technology used during this experiment was GreenPower LEDs Toplight version 1.2 Deep Red/White/Low Blue High Output (Signify, Eindhoven, The Netherlands), with a spectral quality composition by 88% of deep reed (650 nm), 5% of green (530 nm), and 7% of low blue (460 nm). Fixtures were installed over the plants head and the average photosynthetic photon flux density (PPFD) emitted from the LEDs, at plants apical meristem’s height, was 168 µmol m^−2^ s^−1^ (10.9 mol m^−2^ d^−1^, considering a SL period of 18 hours). SL treatment started 120 DAT and it was stopped 243 DAT, when the DLI of natural light in the glasshouse was above 25 mol m^−2^ d^−1^. The maximum photoperiod used during the experiment was of 18 hours and it was regulated according to the amount of natural light supplied by the sun [9]. To measure PPFD and DLI in the glasshouse, a quantum sensor (LI-191SA, LI-COR Biosciences, Superior Street Lincoln, NE, USA) was placed at the height of the tomato plants’ heads.

### 2.4. Yield and Average Fruits Weight 

Seven DAT, six plants per each cultivar in three blocks were marked in both greenhouse compartments (natural light vs. natural light + LEDs). The measurements were made every seven days on these plants, until the end of the cycle, and the considered parameters were: fruit weight, number of fruits per truss, and number of harvested trusses. Yield was calculated every week for each plant, with the following formula: [(Average fruit weight) × (Average fruit number per truss) × (Number of harvested trusses)].

### 2.5. Dry Weight, Total Soluble Solids, pH and Tritratable Acidity Measurements

Qualitative analyses were conducted on a sample for each genotype, for the three replicates, for both light conditions harvested on 180 DAT (sixty days after that LEDs were switched on). For TSS measurement, three tomatoes per elementary experimental unit and per harvest were randomly selected and TSS content was determined using a portable reflectometer (Brixstix BX 100 Hs; Techniquip Corp., Livermore, CA, USA). The dry weight (DW) was measured in triplicate by oven-drying at 65 °C until a constant weight of the samples. Tritratable acidity (TA) was determined by titrating diluted tomato product to pH 8.00 with 0.1 N NaOH and it was expressed in terms of citric acid concentration. Before the titrating operation, initial pH was determined for diluted tomato product.

### 2.6. Vitamin C and Total Antioxidant Activity 

Tomato berries (5 g) were ground in a waring blender for 2 min using liquid nitrogen and stored at −80 °C until analysis. Tomato homogenized samples were mixed with 6% meta-phosphoric acid (1:4, *w*:*v*) and incubated for 30 min at 4 °C for hydrophilic fraction extraction; after centrifugation at 20,000× *g* for 15 min, supernatants were collected and used for vitamin C analysis according to Paradiso et al. [31].

Tomato fruit homogenate was freeze dried by using a freeze dryer (ScanVac CoolSafe 55–9 Pro LaboGene ApS, Lynge, Denmark) and then ground in a laboratory ultra-centrifugal mill (ZM200, Retsch GmbH, Haan, Germany) through 500 µm and used for antioxidant activity assay. Trolox or α-tocopherol equivalent antioxidant capacity (TEAC or α-TEAC) were measured for the hydrophilic or hydrophobic fractions respectively using the ABTS decolorization assay according to Re et al. [32] with modifications. Briefly, extraction of hydrophilic fraction was accomplished by mixing freeze-dried tomato powder with 6% metaphosphoric acid (1:10, *w*:*v*) as described above; lipophilic fraction was extracted from the resulting pellet with a solution of Hexane: Acetone: MET-OH (2:1:1) in a 1:5 ratio (*w*:*v*) for 2 h at 4 °C in continuous agitation. Hydrophilic and lipophilic phases were collected after centrifugation at 20,000× *g* for 15 min at 4 °C. ABTS^+^ stock solution was prepared by incubating overnight in the dark 7 mM ABTS and 2.45 mM potassium persulfate in water. The hydrophilic fractions were diluted 1:10 in PBS and mixed with diluted ABTS^+^ (A_734_ = 0.7) solution in PBS (50 µL samples or Trolox standard in 950 µL ABTS^+^). Difference of absorbance at 734 nm was measured after 5 min of incubation at 25 °C, by means of a spectrometer (Shimadzu UV-1800, Kyoto, Japan). TEAC values were calculated from the Trolox standard curve (0–300 µM). The hydrophobic fractions were mixed with diluted ABTS^+^ (A_734_ = 0.7) solution in EtOH (50 µL samples or α-tocopherol standard in 950 µL ABTS^+^). Difference of absorbance at 734 nm was measured after 30 min of incubation at 25 °C. α-TEAC values were calculated from the α-tocopherol standard curve (0–300 µM). All measurements were carried out in triplicate.

### 2.7. Carotenoids and Tocopherols Analysis

Triplicate aliquots of freeze-dried tomato powder (500 mg) were resuspended in 5 mL of distilled water obtaining a homogeneous suspension. Carotenoids and tocopherols extraction was performed on 500 mg of the tomato homogeneous suspension as reported in Palmitessa et al. [33]. Quantitative analyses of carotenoids and tocopherols were carried out by HPLC as described by Durante et al. [14].

### 2.8. Elemental Analysis

Macro and microelements (Ca, K, Mg, Na, Al, B, Fe, Mn, Mo, Cu, and Zn) concentrations were determined according to D’Imperio et al. [34]. Briefly, 0.30 g of dried sample were mineralized in 10 mL of 65% HNO**_3_** (Pure grade, Carlo Erba, Cornaredo, Milano, Italy) in microwave digestion system (MARS 6, CEM Corporation, Matthews, NC, USA). The digestion procedure was performed in two steps: 15 min to reach 200 °C and 10 min maintained at 200 °C (power set at 900–1050 W; 800 psi). The digested solutions were cooled and quantitatively transferred to a 50 mL volumetric flask. Each solution was diluted to volume with ultrapure H_2_O (Milli-Q Millipore 18 M Ω/cm) and filtered using a 0.45 µm filter.

Samples were analyzed with a inductively coupled plasma-optical emission spectrometry (ICP-OES; 5100 VDV, Agilent Technologies, Santa Clara, CA, USA) to measure Ca, K, Mg, and Na in radial mode and Al, B, Fe, Mn, Zn, and Cu in axial mode [35].

### 2.9. Statistical Analysis

All data underwent analysis of variance (ANOVA) using the General Linear model (GLM; SAS Software, Cary, NC, USA). The experimental factors were considered fixed and processed by two-way analysis of variance (ANOVA) with a nested experimental design and three replicates; the orthogonal contrasts technique was used to establish the differences between cultivar means (two contrasts): (1) RC vs. (YP and RP); (2) YP vs. RP.

## 3. Results

### 3.1. Supplemental Light Treatment

Tomato plants were transplanted in late summer and LEDs were switched on 120 DAT (mid-December). From 121 DAT to 243 DAT the amount of total DLI (DLI_NL+SL_) supplied to the tomato plants increased from 19.73 mol·m^−2^·d^−1^ to 30.16 mol·m^−2^·d^−1^ (Table 1). This was due to the increase of natural DLI (DLI_NL_) from December to the end of the experiment (Table 1). Consequently, the relative (%) amount of DLI supplied from LEDs (DLI_SL_) decreased from 48%, between 121 and 150 DAT, to 8% between 211 and 243 DAT.

### 3.2. Yield and Average Fruits Weight

The average fruits weight did not change between LEDs and NL conditions, but it was affected by cultivars (Table 2). RC had an average fruit weight value 4% higher than YP and RP, while YP showed an average fruit weight value 5% higher than RP (Table 2). Finally, plants grown under LEDs reached an average yield by 21% higher than the plants grown with NL (Table 2). On detail, YP and RP had 28% higher yield than RC and considering the two tomato plum typologies, the yellow one (YP) had 14% higher yield than the red one (Table 2).

### 3.3. Dry Weight, Total Soluble Solids, pH, and Titratable Acidity Measurements

**Figure 1:**
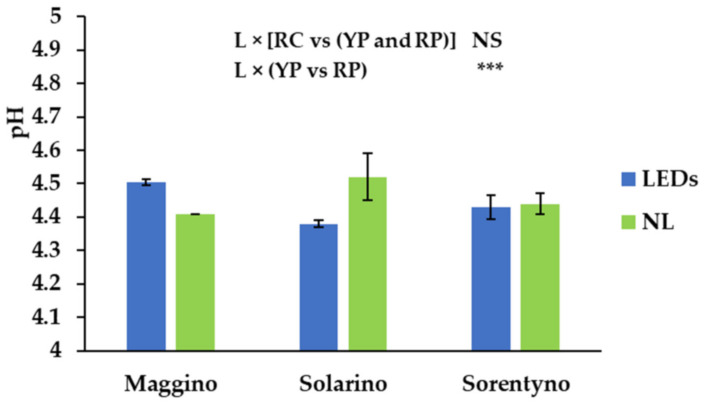
pH values of three tomato genotypes grown under natural light (NL) or supplemental LED light (LEDs): “Maggino” (YP), “Solarino” (RP), and “Sorentyno” (RC). Vertical bars indicate ± SE. Significance: *** for *p* < 0.01; NS not significant.

Tomato fruits grown under LEDs had 3% more of dry matter compared to the fruits grown only under NL (Table 2). Considering the genotypes, RC tomato fruits contained almost 14% more dry matter than the plum tomatoes; between the latter two, the red one had 9% more dry matter than the yellow one (Table 2). Same trend was observed measuring TSS (Table 2). This parameter reached the highest values when tomato plants were grown under LEDs (Table 2). It was almost 15% higher under LEDs than under NL and for cherry tomato genotype it was almost 5% higher than plum genotypes, while, considering only the plum tomato typology, red plum had almost 9.2% higher TSS than yellow plum (Table 2). When YP grown under LEDs, the pH values were 2% higher than those of fruits grown with NL; differently, pH of “Solarino” was 2.2% more under NL than under LEDs (4.50 vs. 4.40; Figure 1). Finally, considering the titratable acidity, expressed as grams of citric acid per 100 mL in the juice, it was almost 16% higher for the tomato fruits grown under LEDs (Table 2). As found for dry matter content and TSS, cherry tomatoes showed almost 12% more titratable acidity than plum genotypes (Table 2).

### 3.4. Vitamin C and Total Antioxidant Activity

**Figure 2:**
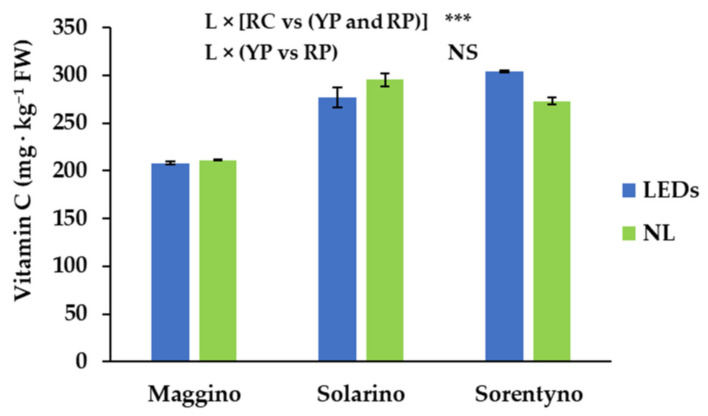
Vitamin C content of three tomato genotypes grown with natural light (NL) or supplemental LED light (LEDs): “Maggino” (YP), “Solarino” (RP), and “Sorentyno” (RC). Vertical bars indicate ± SE. Significance: *** for *p* < 0.001; NS not significant.

YP had the lowest vitamin C content (Table 3). YP and RP did not show significant differences growing under different light conditions (Figure 2). Differently, when “RC” was grown under LEDs, vitamin C content was, on average, 304 mg kg^−1^ FW, almost 15% higher than under NL (Figure 2). Total antioxidant activity was more than ten times higher in hydrophilic fraction than in lipophilic (Table 3). In detail, the lipophilic fraction was almost 8% higher for tomato fruits cultivated under LEDs than under only NL; while, between the genotypes, the red cherry one (RC) had almost 16% less antioxidant activity in lipophilic fraction than red and yellow plum genotypes (RP and YP; Table 3). No difference between light treatments was found in the tomato fruits referred to as the antioxidant activity in hydrophilic fraction but, considering the cultivars, the fruits of YP showed 21% less antioxidant activity in hydrophilic fraction compared to RP (Table 3). Finally, RC had 19% lower antioxidant activity in hydrophilic fraction than red and yellow plum genotypes (Table 3).

### 3.5. Analysis of Carotenoids and Tocopherols

**Figure 3:**
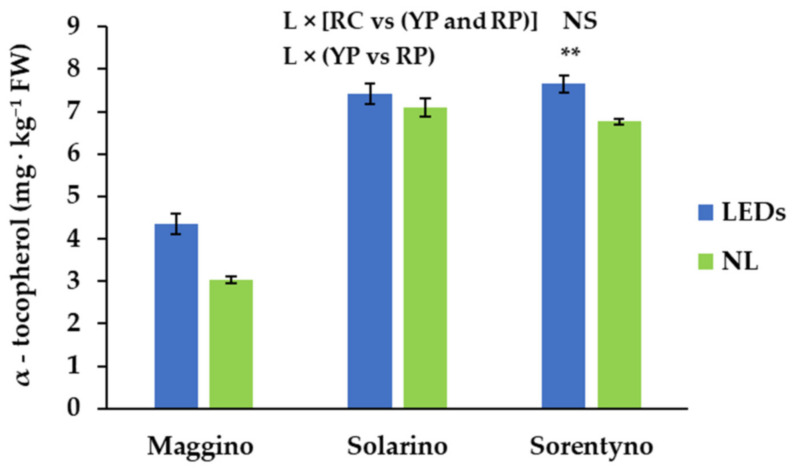
α-tocopherol content of three tomato genotypes grown with natural light (NL) or supplemental LED light (LEDs): “Maggino” (YP), “Solarino” (RP), and “Sorentyno” (RC). Vertical bars indicate ± SE. Significance: ** for *p* ≤ 0.01; NS not significant.

When tomato plants were grown under SL, fruits had almost 15% higher α-tocopherol content compared to the fruits harvested in NL (Table 4). On average, RC showed almost 32% higher α-tocopherol than plum tomatoes, while red plum tomatoes had 3.56 mg of α-tocopherol kg^−1^ FW more than yellow plum fruits (Table 4). The α-tocopherol content of RP did not vary between light treatments, while when YP and RC were grown under LEDs the α-tocopherol content of tomato fruits increased by 43% and 13%, respectively, compared to NL condition (Figure 3).

**Figure 4:**
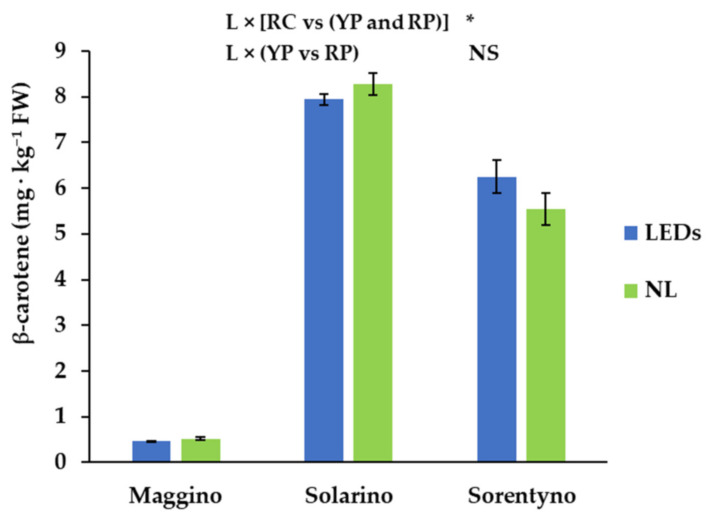
β-carotene content of three tomato genotypes grown with natural light (NL) or supplemental LED light (LEDs): “Maggino” (YP), “Solarino” (RP), and “Sorentyno” (RC). Vertical bars indicate ± SE. Significance: * *p* < 0.05; NS not significant.

*Trans*-lycopene was the most abundant carotenoid found in red tomato fruits, while in yellow tomato the most abundant carotenoids were lutein and β-carotene (Table 4). YP had the lowest carotenoid content: its fruits had around half content of lutein and 18.5 mg *trans*-lycopene kg^−1^ FW less than RP (Table 4). Moreover, the content of *trans*-lycopene was almost 9% higher in cherry tomato than red plum tomato fruits (Table 4). Although zeaxanthin is present in lower amount than other carotenoids identified in tomato fruit samples, YP was the genotype with the highest zeaxanthin content (Table 4). The average zeaxanthin content found in yellow plum tomato fruits (for whatever light condition) and red plum tomato fruits (only when plants were grown with LEDs) was 0.054 mg kg^−1^ FW (Table 4). Contrary to zeaxanthin content, the highest content of β-cryptoxanthin was found in cherry tomato genotype (Table 4). In fact, in RC, on average, 3.2 mg of β-cryptoxanthin kg^−1^ FW more than RP and YP was found (Table 4). Finally, the second most abundant carotenoid in red tomato fruits was β-carotene (Table 4); on average, YP showed a value 17 and 12 times lower than RP and RC, respectively. Furthermore, β-carotene content increased in RC when plants were grown under LEDs, while this trend was not observed for RP and YP (Figure 4).

### 3.6. Mineral Analysis

**Figure 5:**
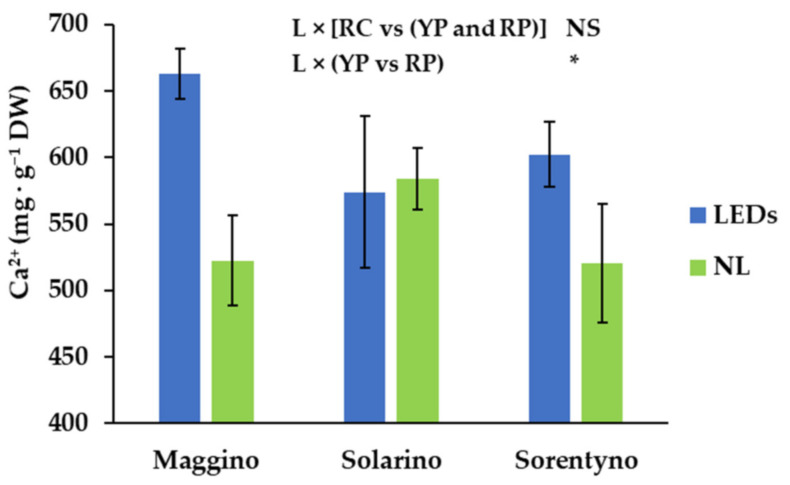
Calcium (Ca^2+^) content of three tomato genotypes grown with natural light (NL) or supplemental LED light (LEDs): “Maggino” (YP), “Solarino” (RP), and “Sorentyno” (RC). Vertical bars indicate ± SE. Significance: * *p* < 0.05; NS not significant.

The most abundant cations in tomato fruits were in the order: potassium (K^+^) > magnesium (Mg^2+^) > calcium (Ca^2+^) > sodium (Na^2+^) > iron (Fe^2+^) > zinc (Zn^2+^). K^+^ was always higher than 40 g kg^−1^ DW (Table 5). They were not influenced by light treatment, but yellow plum tomatoes had almost 15% more K^+^ than the red ones (Table 5). Mg^2+^ content was influenced by light treatment and genotype; in fact, the tomato fruits grown under LEDs showed almost 12% more Mg^2+^ than NL condition (Table 5). In addition, cherry tomato genotype had almost 21% higher Mg^2+^ content than the two plum genotypes (Table 5). On average, Ca^2+^ was 579 mg kg^−1^ DW, but the interaction between light conditions and the plum genotypes showed a significant variation on the content of this cation: when YP was grown under LEDs, fruits had almost 27% of Ca^2+^ content higher than the same fruits obtained without LEDs, while for RP Ca^2+^ content did not vary between light treatments (Figure 5).

On average, sodium (Na^2+^) was 142 mg kg^−1^ DW, while Fe^2+^ content varied significantly between the two plum genotypes (Table 5). In fact, RP had almost 2.3 times the Fe^2+^ content of YP (Table 5). Regarding molybdenum (Mo^2+^), its concentration was 50% higher in the tomato fruits grown under LEDs than under NL condition, and the fruits of YP had more than double of Mo^2+^ than RC and RP (Table 5). Manganese (Mn^2+^) was almost 24% higher in red plum tomato fruits than in yellow plum (Table 5). Finally, about aluminum (Al^3+^), boron (B^3+^), copper (Cu^2+^), and zinc (Zn^2+^), their average contents in tomato fruits were 14, 9.5, 12, and 25 mg kg^−1^ DW, respectively.

## 4. Discussion

In this work, we performed the growth of three different hybrids of tomato using a soilless system in a Southern Italian greenhouse during the winter–spring seasons, supplementing the NL with deep red/white/low blue LEDs light. The study was conducted on red cherry type, red plum type, and yellow plum type tomato fruits because these are the most appreciated commercial typologies by the Italian consumers. During the four-months light treatment (from 121 to 243 DAT), SL was on average 30% of NL (Table 1). Tomato plants had an average yield of 8.0 kg m^−2^ with SL treatment and 5.8 kg m^−2^ under LEDs and NL [9]. In detail, for RP and YP, 7.3 and 5.1 kg m^−2^ of tomato fruits were harvested for SL and NL treatment, respectively; while, during the same period, RP yielded 8.7 kg m^−2^ under LEDs and 6.4 kg m^−2^ under NL conditions [9]. After the results obtained about yield, in this experiment we investigated if LEDs supplementary light source-maintained tomato fruit quality in Mediterranean greenhouse climatic conditions. Previously, through three experiments conducted in a greenhouse in West Lafayette, (IN, 40° N, 86° W), Dzakovich et al. [35] found that LEDs, as source of SL, did not affect greenhouse tomato fruit quality.

Analyzing DW and TSS content (Table 2), YP had the lowest values (9.2 °Brix), similarly to the results obtained by Palmitessa et al. [33]. Since soluble sugars and organic acids represent 50% and 15% of the total fruit DW [36], respectively, we can confirm that this yellow plum tomato hybrid has a particularly low content of these substances compared to the other genotypes. However, considering that the TSS of tomato fruits ranged between 3.59% and 4.40% [23], YP had an appreciable TSS content (Table 2). Moreover, when the three tomato hybrids were grown under SL, they increased the DW and TSS content of tomato fruits (Table 2), similarly to the results obtained by Jiang et al. [37] in Japan (34°53′29.5″ N, 139°65′14.1″ E). They found that the three leaves under the fruit truss mostly contributed to fruit production and, by increasing their photosynthesis activity, enhanced fruit DW and TSS [37]. Instead, the pH of the tomato fruits did not vary between the cultivars and between light conditions; only for RP, when the plants were grown under LEDs, the pH decreased, probably because the organic acid content increased considerably (Table 2). In fact, the titratable acidity was higher for the plants grown under LEDs than those grown only under natural light (Table 2). Titratable acidity is used as an indicator of fruits maturity [23]: its content increased when tomato plants were grown under LEDs [35] and/or when LEDs artificial light was applied as the postharvest technology [38].

Vitamin C content of the three cultivars grown under NL are in good agreement with the previous studies [33]. YP showed the lowest vitamin C content, while RP and RC showed similar values (Table 3). Moreover, Loi et al. [39] reported that vitamin C synthesis is light dependent and its synthesis depends on light intensity, quality, and penetration into the canopy. During this study, the vitamin C content was influenced by the interaction of SL treatment with genotypes (Figure 2). In fact, it was not negatively affected in both YP and RP under SL, while it was even enhanced in RC grown under SL, being almost 15% higher than under NL (Figure 2). Probably, the different plant architecture determined a different fruit exposition to SL radiation and for this reason RC increased ascorbic acid content when it was grown under LEDs, while this was not found for YP and RP. A similar result to that obtained for RC was found by Gautier et al. [40] for red cherry tomatoes, with SL directed to the whole fruits.

Total antioxidant activity of the hydrophilic fraction is in rough agreement with a previous study for YP and RP and is significantly lower for RC [33]. (Table 3). Such a difference could be attributed to the different seasonal period during which the growth took place. In any case, all fruits showed the same antioxidant activity when grown under NL or SL, indicating a similar level of hydrophilic antioxidant compounds. Conversely, the antioxidant activity of the lipophilic fraction, mainly attributed to the carotenoid and tocopherol contents, was significantly higher in tomato fruits cultivated under SL than those grown with only NL. This result can be explained by the increased amount of α-tocopherol found under SL. A previous study reported that blue, red, and far red light increased tomato fruit amount of both lycopene and β-carotene by stimulating the phytochrome activity [41]. However, our results indicate that SL treatment did not significantly affect carotenoid content, as reported also by Dzakovich et al. [35], with the only exception of zeaxanthin. This is a very important result, because the biosynthesis of lycopene is negatively affected by some environmental conditions, as high sunlight irradiation level [42], while during this experiment the supplemental radiation supplied by LEDs increased tomato yield [9] and did not reduce carotenoid content (Table 4).

In agreement with the results obtained by Palmitessa et al. [33] and Raiola et al. [43] α-tocopherol, the most biologically active form of vitamin E, showed higher levels than β-tocopherol (Table 4). Its contents ranged from 3.79 to 7.25 mg kg^−1^ FW, similarly to the values obtained by Caretto et al. [21], while β-tocopherol was only detected in RP (Table 4). As described for vitamin C, the effects of SL treatment on α-tocopherol content were genotype specific (Figure 3).

As outlined in Table 5, it was not possible to define a common trend for the mineral tissue content in relation to the light treatments, since the mineral profile was different according to the genotype and the light conditions. In general, different tomato genotypes showed different mineral profiles, as reported here and in another study [44]; the major differences between the three cultivars found in this current study was the high content of Fe in RP, probably related to the best capacity of this genotype to uptake this element. It is interesting to underline that light played an important role in the enhanced uptake of Ca^2+^ in YP and Mg^2+^ and Mo^2+^ in all genotypes. Similar results were found in lettuce [45]: the light spectrum used to produce vegetables modified the uptake of Ca^2+^, Mg^2+^ and other minerals, macro and microelements. This is a relevant result, because Ca^2+^ and Mg^2+^ are important mineral elements for consumers, being essential structural components in bones and teeth, taking part in different physiological and biochemical processes. In this context, it is important to highlight that in literature different biofortification studies were performed with the aim of increasing the Ca^2+^ content in edible parts of different vegetables by using agronomic and transgenic approaches [46,47]. Our results indicate that the supplemental light technology could be used as an alternative and innovative method for producing tomato fruits biofortified with Ca^2+^. Tomato with a higher mineral concentration would allow consumers to improve the intake of minerals without requiring an increase in daily consumption.

## 5. Conclusions

Red plum tomatoes (“Solarino”; RP), red cherry tomatoes (“Sorentyno; RC”), and yellow plum tomatoes (“Maggino; YP”), the three hybrids studied in this experiment, are the most abundant commercial typologies of fresh tasty tomatoes consumed in Italy. Moreover, LEDs technology is increasing its application in Mediterranean greenhouse conditions, thus, based on the results obtained during our previous research [9] and the results obtained during this study, we can conclude that deep red/white/low blue LEDs, used as supplementary light system in an innovative semi-closed greenhouse in Mediterranean area:

- Increased tomato fruit production;
- Maintained the antioxidant property of the hydrophilic fraction and increased that of the lipophilic fraction as well as the α-tocopherol content (particularly for yellow plum and cherry tomato types);
- Maintained or increased (depending on the tomato hybrids) the mineral profile of the tomato fruits;
- Increased DW, TSS, and TA of tomato fruits;
- Could be used as an innovative method for producing tomato fruits biofortified with Ca^2+^ and Mg^2+^.

## Figures and Tables

**Table 1:** Daily light integral (DLI) supplied from natural light (DLI_NL_), LEDs (DLI_SL_) and their sum (DLI_NL + SL_) during different period of crop cycle.

Day after Transplant (DAT)	Natural Light (DLI_NL_)	LEDs (DLI_SL_)	Total (DLI_NL + SL_)	% SL
	(mol m^−2^ d^−1^)			
121–150	10.21	9.52	19.73	48
151–180	14.69	8.91	23.60	38
181–210	20.63	6.57	27.20	24
211–243	27.70	2.46	30.16	8

**Table 2:** Main effects of light treatment and genotypes on average fruit weight, yield, dry weight (DW), total soluble solids (TSS), pH, and titratable acidity.

	Average Fruit Weight	Yield	Dry Weight (DW)	TSS	pH	Titratable Acidity
	g	g·Plant^−1^	(g·100 g^−1^ FW)	(°Brix)		(g of Citric Acid·100 mL^−1^)
**Light (L)**						
LEDs	8.5 ± 0.8	4684 ± 150	10.4 ± 0.8	10.8 ± 0.8	4.44 ± 0.06	0.64 ± 0.06
Natural Light	8.4 ± 0.8	3865 ± 96	10.1 ± 0.7	9.4 ± 0.6	4.46 ± 0.08	0.55 ± 0.05
**Cultivar (CV)**						
Maggino (Y)	8.5 ± 0.4	4801 ± 130	9.5 ± 0.3	9.2 ± 0.6	4.46 ± 0.05	0.58 ± 0.08
Solarino (P)	8.1 ± 0.3	4220 ± 102	10.2 ± 0.4	10.7 ± 0.9	4.45 ± 0.11	0.56 ± 0.07
Sorentyno (C)	8.6 ± 0.4	3517 ± 89	11.1 ± 0.1	10.4 ± 0.6	4.44 ± 0.05	0.64 ± 0.03
Significance ^1^						
L	NS	***	**	***	NS	***
CV	*	***	***	***	NS	*
C × (Y and P)	*	***	***	**	NS	*
Y vs P	**	***	**	***	**	NS
L × CV	NS	NS	NS	NS	*	NS
L × [(Y and P) vs C]	NS	NS	NS	NS	NS	NS
L × (Y vs P)	NS	NS	NS	NS	NS	NS

^1^ Significance: ***, **, and *, respectively, for *p* ≤ 0.001, *p* ≤ 0.01, and *p* ≤ 0.05; NS, not significant. The significant interaction is shown in Figure 1.

**Table 3:** Main effects of light treatment and genotypes on the ascorbic acid content and the total antioxidant activity (lipophilic and hydrophilic fraction).

		Total Antioxidant Activity	
	Vitamin C	Lipophilic Fraction	Hydrophilic Fraction
	(mg·kg^−1^ FW)	(TEAC meq·kg^−1^ FW)	
**Light (L)**			
LEDs	263	115	1432
Natural Light	260	106	1376
**Cultivar (CV)**			
Maggino (YP)	210	107	1354
Solarino (RP)	286	122	1628
Sorentyno (RC)	288	101	1249
Significance ^1^			
L	NS	**	NS
CV	***	*	*
RC vs (YP and RP)	***	*	*
YP vs RP	***	NS	*
L × CV	**	NS	NS

^1^ Significance: ***, **, and *, respectively, for *p* ≤ 0.001, *p* ≤ 0.01, and *p* ≤ 0.05; NS = not significant. The significant interaction is shown in the Figure 2.

**Table 4:** Main effects of light treatment and genotypes on α-tocopherol, lutein, zeaxanthin, β-cryptoxanthin, β-carotene, and trans-lycopenes.

	α-Tocopherol	β-Tocopherol	Lutein	Zeaxanthin	β-Cryptoxanthin	β-Carotene	*Trans*-Lycopene
	(mg·kg^−1^ FW)						
**Light (L)**							
LEDs	6.47	3.85	0.86	0.04	1.22	4.89	13.5
Natural Light	5.63	4.04	0.83	0.02	1.15	4.78	13.1
**Cultivar (CV)**							
Maggino (YP)	3.69	0.00	0.55	0.06	0.11	0.49	0.5
Solarino (RP)	7.25	3.95	1.10	0.03	0.11	8.11	19.0
Sorentyno (RC)	7.21	0.00	0.88	0.00	3.33	5.89	20.8
Significance ^1^							
L	**	NS	NS	**	NS	NS	NS
CV	***	***	***	***	***	***	***
RC vs (YP and RP)	***	***	NS	***	***	***	***
YP vs RP	***	***	***	***	NS	***	***
L × CV	NS	NS	NS	***	NS	NS	NS

^1^ Significance: *** and **, respectively, for *p* ≤ 0.001 and *p* ≤ 0.01; NS not significant. The significant interactions are shown in the Figure 3 and Figure 4.

**Table 5:** Main effects of light treatment and genotype on mineral content of tomato fruits.

	Ca	Fe	K	Mg	Al	B	Cu	Mn	Mo	Na	Zn
	(mg·kg^−1^ DW)										
**Light (L)**											
LEDs	613	139	44,192	1059	14.40	9.80	12.80	12.90	3.60	132	26.40
Natural Light	545	106	44,530	944	13.80	9.10	11.50	12.90	2.40	152	24.20
**Cultivar (CV)**											
Maggino (YP)	592	58	46,425	952	13.30	8.50	13.10	11.50	4.60	129	27.90
Solarino (RP)	579	193	40,442	942	11.20	12.10	9.80	14.30	2.10	161	24.60
Sorentyno (RC)	569	118	46,552	1143	18.70	7.60	14.00	12.90	2.30	134	23.20
Significance ^1^											
L	NS	NS	NS	**	NS	NS	NS	NS	*	NS	NS
CV	NS	*	*	***	NS	NS	NS	*	**	NS	NS
RC vs. (YP and RP)	NS	NS	NS	***	NS	NS	NS	NS	*	NS	NS
YP vs RP	NS	*	*	NS	NS	NS	NS	*	**	NS	NS
L × CV	*	NS	NS	NS	NS	NS	NS	NS	NS	NS	NS

^1^ Significance: ***, **, and *, respectively, for *p* ≤ 0.001, *p* ≤ 0.01, and *p* ≤ 0.05; NS not significant. The significant interaction is shown in the Figure 5.

## Data Availability

The raw data supporting the conclusions of this article will be made available by the authors, without undue reservation.
